# Delirium management and current practice among Intensive Care Units Doctors, Khartoum

**DOI:** 10.12688/f1000research.142233.2

**Published:** 2024-10-31

**Authors:** Sheema Hamid Seidna Hamid, Ghada Omer Hamad Abd El-Raheem, Hana Eltayeb Salih Elamin, Mudawi Mohammed Ahmed Abdallah

**Affiliations:** 1 Clinical Pharmacy, Omdurman Islamic University, Omdurman, Khartoum, 11115, Sudan; 2 Clinical pharmacy, Soba University Hospital, Khartoum, Khartoum, 11111, Sudan; 3 Research methodology and Biostatistics, University of Medical Sciences and Technology, Khartoum, Khartoum, 11111, Sudan; 4 Medical Manager of Critical Care, Military Hospital, Omdurman, Khartoum, 11115, Sudan

**Keywords:** Delirium, CAM-, ICDSC, critically ill patients, ICU, current practice, Intensive Care Units, Doctors.ICU

## Abstract

Delirium is a brain dysfunction characterized by attention and cognitive disturbances in a fluctuating manner. The international guidelines recommend daily screening for delirium. The Confusion Assessment Method for the Intensive Care Unit (CAM-ICU) and Intensive Care Delirium Screening Checklist (ICDSC) are the most commonly used methods for assessing delirium. This study aimed to identify barriers and gaps in knowledge and practice. This was a hospital-based Cross-Sectional study. Stratified random sampling was used in this study. 72 ICU doctors were randomly selected. Statistical analyses were performed using IBM SPSS version 23. Descriptive data were presented, and the chi-squared test was used to determine the associations among variables. Statistical significance was set at *p* < 0.05. More than 70% of the doctors were ≤ 30 years of age and female. A total of 69.4% of the participants had < 1year of experience. In total, 94.4% of the participants worked in medical ICUs. Less than 20% of the doctors used delirium assessment tools, with a statistically significant difference based on experience ( *p*=0.012). Delirium was not regularly assessed in 13.9% of the patients. Non-pharmacological management was applied by 76.4% of doctors, and communication with patients was the most frequent (75%). Haloperidol was the most commonly used drug (83.3%). A total of 40.3% of doctors did not stop delirium medication on ICU discharge. A regular delirium assessment was performed. However, the use of validated assessment tools is uncommon. Nonpharmacological management of delirium is important and is mostly performed. Our doctors prescribed antipsychotics for the treatment of both forms of delirium, and almost half of them did not stop the medications on ICU discharge. Medication reconciliation and contact with the next in-charge of the patients are important.

**Abbreviations**
CAM-ICU: Confusion Assessment Method for the Intensive Care Unit
ICDSC: Intensive Care Delirium Screening Checklist
ICU: Intensive Care Unit
MV: Mechanical Ventilator

## Background

Delirium is a state of brain dysfunction characterized by attention and cognition disturbances in a fluctuating pattern, ^1^^–^^7^ with an acute onset of confusion and decline in cognitive ability, often occurring in hospitalized patients. ^8^ Delirium is related to many factors, such as the pre-existing vulnerable state of patients or medication-induced delirium that occurs during hospital stay. ^9^ Delirium is a common disorder among intensive care unit (ICU) patients because of many factors such as old age, multiple medical interventions, and critical illness severity. ^5^^,^^10^ Critically ill patients who are complicated with delirium, in addition to their critical illness, have been associated with prolonged mechanical ventilation (MV), longer hospital and ICU stays, and increased mortality. ^3^^,^^4^^,^^6^^,^^8^^,^^11^^–^^13^ The severity of adverse delirium outcomes was associated with the duration of delirium; the longer the duration, the worse the outcomes. ^11^ Prolonged delirium in the ICU is considered a risk factor for developing post-intensive care syndrome, which is characterized by worsened or new impairments in cognitive, physical, and mental health. ^12^ Acute delirium can persist for a few hours; however, it can persist for weeks to months after hospital discharge. ^14^ Clinically, delirium can be hyperactive or hypoactive. In the hyperactive form, agitation is prominent, with frequent aggression and risk of self-harm. In the hypoactive form, the patient presents with a low level of consciousness, which is usually prostrate and uncommunicative. A mixed form may also occur with alternation between the two poles. ^14^

Its incidence varies widely; however, it has a high rate of 70–87%. ^3^^,^^6^^,^^8^^,^^11^^,^^12^^,^^15^^,^^16^ Although delirium is common, it is preventable. Prevention or early management of delirium is crucial to reverse the delirium state and minimize adverse clinical outcomes. ^7^^,^^11^

The nature of the underlying critical illness, as well as the lack of any verbal communication among ICU patients, poses a difficulty in delirium assessment in the ICU. ^5^ Furthermore, delirium is associated with adverse consequences such as long-term cognitive impairments. ^8^^,^^17^ Systematic assessment of delirium among ICU patients is considered a very important approach to deliver patient care and allows clinicians and other healthcare staff to detect delirium at an early stage. ^1^^,^^2^

From another perspective, patients with delirium are more likely to bear increased healthcare costs than those without delirium. ^12^^,^^15^^,^^17^

Management of delirium might be challenging for ICU clinicians, as an established treatment plan is yet lacking. ^17^ International guidelines recommend daily screening for delirium using validated delirium assessment tools. ^18^ Several methods have been developed and validated to assess delirium in patients in the ICU. Of these tools, the Confusion Assessment Method for the Intensive Care Unit (CAM-ICU) and Intensive Care Delirium Screening Checklist (ICDSC) are the most commonly used for delirium assessment. ^19^ In the CAM-ICU, four features were screened at a single point in time. The ICDSC has a screening checklist composed of eight features. ^16^^,^^18^ These are the most frequently used tools for delirium screening. ^19^ Furthermore, these instruments could also be used in patients with primary neurological injury, as growing evidence suggests. ^20^

In particular, screening is mandatory for patients at moderate to high risk of delirium. ^18^

Barriers and gaps in practice were addressed by assessing the current status to identify the areas that need focusing on. ^21^ Several studies have focused on delirium assessment by clinicians. ^3^^,^^18^^,^^22^ In Sudan, no studies have been conducted in the ICU, particularly regarding delirium. This study assessed the current status of knowledge and practice of ICU doctors about delirium. The reasons why this study was developed were to identify the barriers and gaps in knowledge and practice among doctors in Sudan. No similar study was done in Sudan and little is known about Sudanese doctors ICU practice for delirium. This study involved military hospitals in Khartoum, however the types of patients involved citizens. Military hospitals provide healthcare services for all types of patients not only military.

## Methods

### Study design

A hospital-based cross-sectional study was conducted to assess the knowledge and practices of intensive care unit doctors at the Military Hospital of Khartoum State, Sudan.

### Setting

The Military Hospital is a complex of seven specialized hospitals totalizing 722 beds and 8 ICUs. The ICUs that met the inclusion criteria were involved in the study; neonatal and maternal ICUs were excluded from the study.

### Participants

At first level, simple random sampling was applied to select the hospitals. Out of the four military hospitals in Khartoum, three hospitals were selected randomly out of all Military hospitals in Khartoum State. At second level, based on the types of ICUs. Three types of ICUs were defined in the military hospitals; surgical, medical and mixed. The stratified random sampling technique was used to select 72 participants proportionally to the type of ICU (68 doctors in medical ICUs, 2 doctors in surgical ICUs and 2 doctors in mixed ICUs). Administrative staff were excluded.

### Variables

The outcome variables were the knowledge and practices of intensive care unit doctors. The related factor to the outcome variable was the years of experience of the participants. Knowledge and practice were assessed through a questionnaire developed by the researchers after reviewing the literature. The questionnaire was then validated by an expert in research methodology ( https://orcid.org/0000-0003-1772-2686). Characteristics of the participants were assessed first, then delirium questions involved assessment tools, first line management, delirium assessment frequency, treatments used and duration of treatment. These predictors for knowledge and practice were important as per the American College of Clinical Pharmacy. ^18^

### Data sources/measurement

The characteristics of the participants were reported as categorical variables; age, gender, ICU type and years of experience. Knowledge and practice of participants were assessed through closed questions entered as categorical variables.

### Bias

Potential source of bias was that the study depended on the self-reporting of the participants through filling the questionnaire.

### Study size

The formula for the known population was used to select the sample size for each stage. The equation of known population was used to estimate the sample size: n=N/1+Nd ^2^, where n is the estimated sample size, N is the total number of doctors in each ICU, and d is the degree of accuracy set at 0.05. All participants had completed the questionnaire with zero refusals. The formula for known population was used to select the sample size at each stage.

### Quantitative variables

All variables included in the study were quantitative; they were handled in the analysis as categorical variables.

### Statistical methods

The Statistical Package for Social Sciences (SPSS version-23) was used to describe and analyze the data. Descriptive data are presented. Statistical analysis was performed using the chi-square test to determine the associations among variables. All tests were considered statistically significant at *p* < 0.05.

The ethics committee of Omdurman Islamic University reviewed and approved the proposal on 28.May.2021 after full board review (IRB name: Omdurman Islamic University Ethics Committee, Reference number: 2021/2). Approval from the Military Hospital was obtained and authorization to implement the research was granted by the administration of the ICUs. All methods were performed in accordance with the relevant guidelines and regulations of the Declarations of Helsinki ( https://www.wma.net/policies-post/wma-declaration-of-helsinki-ethical-principles-for-medical-research-involving-human-subjects/). Participants were informed about the research objectives and signed written informed consent was obtained from each participant prior to data collection. They were assured about their confidentiality through the use of an anonymous research tool and that the data collected from each of them were not to be used for any purposes other than those assigned to the research. Participants were free to accept or reject participation in this study.

## Results

### Characteristics of intensive care unit (ICU) doctors

Seventy-two intensive care unit (ICU) doctors were assessed for their current knowledge and practice towards delirium among critically ill patients. Most doctors (70.8%, 51/72) were aged 30 years or less, and 86.1% (62/72) were female. On assessing experience, 69.4% (50/72) had less than one year of experience. Most doctors (94.4%, 68/72) had been working in medical ICUs. Table 1 below illustrates the demographic characteristics of the study participants.

**Table 1.** Characteristics of ICU doctors (n=72).

Characteristics	n	%
**Age**		
≤30 Years	51	70.8
>30 Years	21	29.2
Total	72	100
**Gender**		
Male	10	13.9
Female	62	86.1
Total	72	100
**Years of experience**		
<1 Year	50	69.4
1-2 Years	7	9.7
>2 Years	15	20.8
Total	72	100
**ICU type**		
Medical ICU	68	94.4
Surgical ICU	2	2.8
Cardiac ICU	2	2.8
Total	72	100

### Assessment of current knowledge and practice of ICU doctors towards delirium

The knowledge and practice of ICU doctors was assessed with regard to delirium. 38.9% of doctors were aware of delirium assessment tools, while 61.1% (44/72) had no knowledge of delirium assessment tools. Of the ICU doctors, 76.4% (55/72) stated that the non-pharmacological approach was their first-line management, whereas 22.2% (16/72) chose the pharmacological approach as shown in Table 2 below.

**Table 2.** Assessment of current practice of ICU doctors about delirium (n=72).

Knowledge and practice	n	%	Knowledge and practice	n	%
**Knowledge about delirium assessment tools**			**Agitation treatment used**		
Aware	28	38.9	Anti-psychotics	39	54.2
Unaware	44	61.1	Sedatives	24	33.3
Total	72	100	Opioids	3	4.2
**Knowledge about first line management**			Antipsychotics+ Sedatives	4	5.6
Non-pharmacological	55	76.4	Do not know	2	2.8
Pharmacological treatment	16	22.2	Total	72	100
Do not know	1	1.4	**Delirium treatment used**		
Total	72	100	Anti-psychotics	50	69.4
**Delirium assessment**			Sedatives	15	20.8
CAM-ICU	13	18.1	Opioids	1	1.4
ICDSC	1	1.4	Antipsychotics+ Sedatives	2	2.8
Signs+ Symptoms	55	76.4	Do not know	4	5.6
None	3	4.2	Total	72	100
Total	72	100	**Stopping delirium medications on ICU discharge**		
**Frequency of delirium assessment**			Yes	41	56.9
Every 8-12 Hours	29	40.3	No	29	40.3
Every 24 Hours	33	45.8	Do not know	2	2.8
Not regularly	10	13.9	Total	72	100
Total	72	100			

As for ICU doctors, the delirium assessment tools used were CAM-ICU (18.1%, 13/72) and ICDSC used by only one doctor. Signs and Symptoms were used by (76.4% (55/72) of the patients for delirium assessment ( Table 2). The frequency of delirium assessment varied among doctors; 40.3% (29/72) of the doctors assessed delirium every 8-12 hours, while 45.8% (33/72) assessed delirium every 24 h. In contrast, 13.9% (10/72) of the doctors did not regularly assess delirium in critically ill patients ( Table 2).

Moreover, the practice of doctors towards delirium management was assessed. More than half of doctors used antipsychotics to manage agitation and delirium (54.2% and 69.4%, respectively). This was followed by sedatives prescribed by 33.3% (24/72) of doctors to treat agitation. For delirium, sedatives were prescribed by 20.8% (15/72) of doctors. Doctors were asked about their practice towards stopping delirium medications for patients on ICU discharge; 56.9% (41/72) of them stopped medications on discharge, while 40.3% (29/72) did not stop them from patients upon ICU discharge ( Table 2).

### Reasons for irregular delirium assessment and not using non-pharmacological approach management among doctors

Ten doctors reported that delirium assessment might be irregular for ICU patients. They were asked to report their reasons for not regularly assessing delirium. Only five doctors mentioned the reasons for this. The first reason was that only after the patient developed signs and symptoms, reported by two doctors, difficulty in assessment was the reason for one doctor, and a high workload was reported by 1doctor. Additionally, the use of family member support was the reason for one doctor.

Doctors were asked to report their reasons for not using the nonpharmacological approach in their delirium management. The lack of knowledge about the non-pharmacological approach was the reason for this.

### Non-pharmacological interventions applied by doctors to reduce delirium

**Figure 1.**
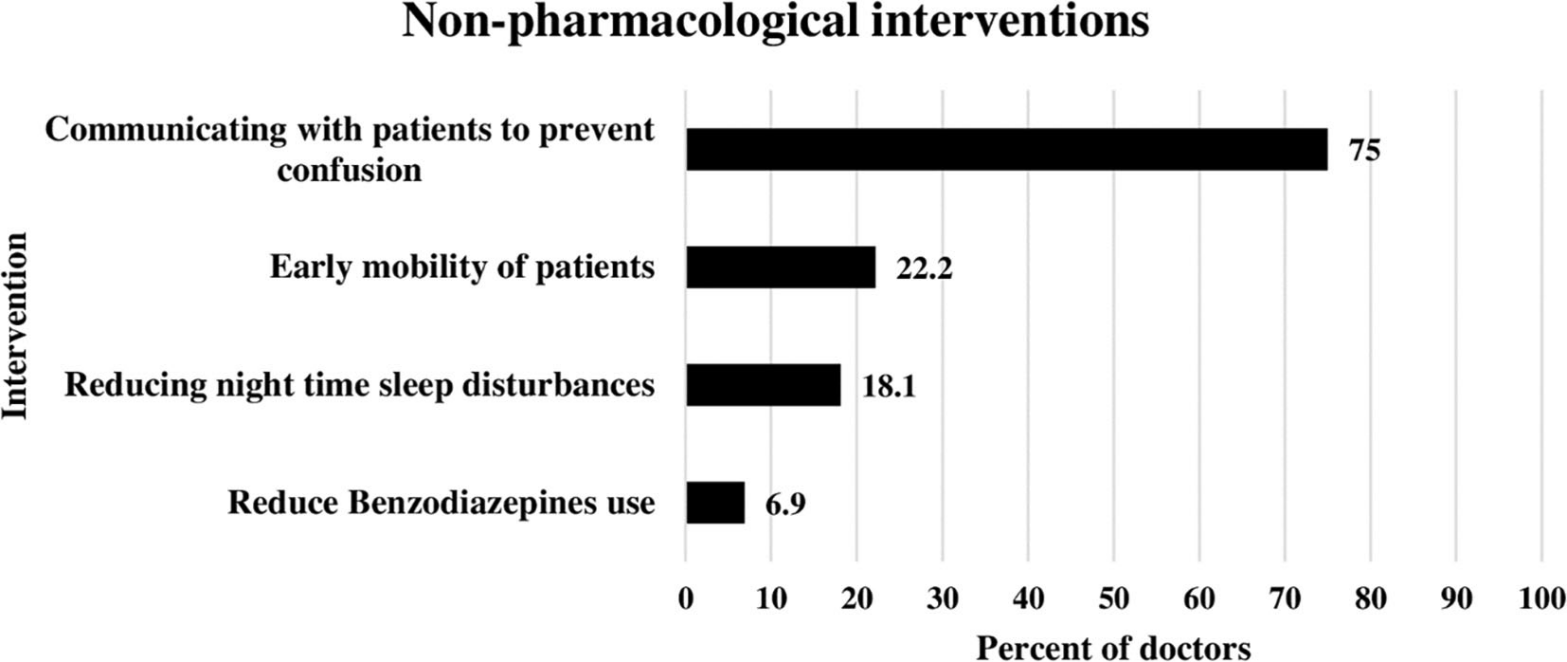
Non-pharmacological interventions applied by doctors to reduce delirium.

The most frequent nonpharmacological intervention used by ICU doctors for delirium management was communication with patients to prevent confusion. This was illustrated in Figure 1 below. This approach was used by 75% of the doctors. The second most common approach was early mobility of ICU patients, reported by 22.2% of doctors. Reducing nighttime sleep disturbances was used by 18.1% of the doctors. Benzodiazepine use was reduced by 6.9% of ICU doctors ( Figure 1).

### Pharmacological agents used by ICU doctors to treat delirium

**Figure 2.**
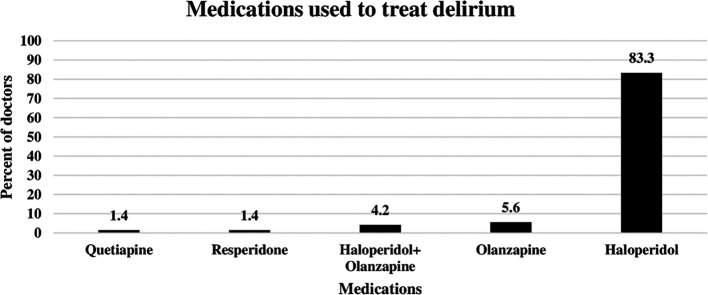
Pharmacological agents used by doctors to reduce delirium.

The doctors were asked to identify the pharmacological agents used for delirium treatment. As shown in Figure 2 below, the most commonly used agent was haloperidol, which was prescribed by 83.3% of the ICU doctors to treat delirium. The second most commonly used agent was olanzapine (5.6%), followed by a combination of olanzapine and haloperidol (4.2%). On the other hand, resperidone and quetiapine were prescribed by only 1.4% of the doctors ( Figure 2).

### Association between knowledge of doctors and their experience

With regard to the experience of doctors, their knowledge was assessed to determine if there was any difference in knowledge about delirium between experienced and non-experienced doctors. Doctors were grouped into two groups based on their years of experience: ≤2 years of experience and >2 years of experience. Interestingly, there was no statistically significant difference in ICU doctors’ knowledge about delirium tools and first-line treatment between experienced and non-experienced doctors ( *p*=0.059 and *p*=0.797, respectively).

Doctors’ practice was assessed based on their experience. Among the 13 doctors who used the CAM-ICU as an assessment tool for delirium, 53.8% had >2 years of experience. Among the 55 doctors who assessed delirium using no tool and only by signs and symptoms, 85.5% had less experience (≤2 years). This difference in practice was statistically significant between experienced and less experienced doctors ( *p*=0.012).

Regarding the frequency of delirium assessment, no statistically significant difference was found between doctors based on their years of experience ( *p*=0.636). In addition, no difference in practice towards agitation and delirium treatment was found among doctors based on their experience ( *p*=0.496 and *p*=0.246, respectively). In addition, stopping delirium treatment at ICU discharge did not differ between doctors with different years of experience ( *p*=0.121). Table 3 below details all the percentages of doctors with different experiences and practices towards delirium.

**Table 3.** Association between knowledge and practice about delirium and years of experience among doctors.

	Years of experience							
Knowledge and practice	≤2 Years	%	>2 Years	%	Total	%	Chi ^2^	*p-*value
**Knowledge about delirium tools**								
Aware	19	67.9	9	32.1	28	38.9	3.55	0.059
Unaware	38	86.4	6	13.6	44	61.1		
Total	57	79.2	15	20.8	72	100.0		
**First-line management**								
Non-pharmacological	44	80.0	11	20.0	55	76.4	0.455	0.797
Pharmacological treatment	12	75.0	4	25.0	16	22.2		
Do not know	1	100.0	0	0.0	1	1.4		
Total	57	79.2	15	20.8	72	100.0		
**Delirium assessment**							10.961	0.012 *
CAM-ICU	6	46.2	7	53.8	13	18.1		
ICDSC	1	100.0	0	0.0	1	1.4		
By signs and symptoms	47	85.5	8	14.5	55	76.4		
None	3	100.0	0	0.0	3	4.2		
Total	57	79.2	15	20.8	72	100.0		
**Frequency of delirium assessment**								
Every 8-12 Hours	22	75.9	7	24.1	29	40.3	0.906	0.636
Every 24 Hours	26	78.8	7	21.2	33	45.8		
Not regularly	9	90.0	1	10.0	10	13.9		
Total	57	79.2	15	20.8	72	100.0		
**Agitation treatment**								
Anti-psychotics	31	79.5	8	20.5	39	54.2	3.381	0.496
Sedatives	17	70.8	7	29.2	24	33.3		
Opioids	3	100.0	0	0.0	3	4.2		
Antipsychotics+ Sedatives	4	100.0	0	0.0	4	5.6		
Do not know	2	100.0	0	0.0	2	2.8		
Total	57	79.2	15	20.8	72	100.0		
**Delirium treatment**								
Anti-psychotics	41	82.0	9	18.0	50	69.4	5.427	0.246
Sedatives	9	60.0	6	40.0	15	20.8		
Opioids	1	100.0	0	0.0	1	1.4		
Antipsychotics+ Sedatives	2	100.0	0	0.0	2	2.8		
Do not know	4	100.0	0	0.0	4	5.6		
Total	57	79.2	15	20.8	72	100.0		
**Stopping delirium medications on ICU discharge**							4.229	0.121
Yes	29	70.7	12	29.3	41	56.9		
No	26	89.7	3	10.3	29	40.3		
Do not know	2	100.0	0	0.0	2	2.8		
Total	57	79.2	15	20.8	72	100.0		

* Statistically significant.

## Discussion

Awareness about delirium assessment in ICU is considered a milestone. Delirium is common disorder among ICU patients. ^3^^,^^6^^,^^8^^,^^11^^,^^12^^,^^15^^,^^16^ Doctors and nurses were the direct healthcare providers for critically ill patients. In addition to nurses, doctors were involved in delirium assessment and reporting as mentioned by Pisani MA et al. ^5^ Lack of awareness among staff was reported as one of the individual barriers. ^3^ An issue to be highlighted in this study was the lack of awareness of Sudanese doctors about delirium assessment tools. Moreover, the ICU staff doctors are mostly young (< 30 years) and inexperienced (< 2 years). This study had put light on that point about the working staff in governmental hospitals in Sudan.

Depending only on signs and symptoms to assess delirium was a common practice among Sudanese doctors. This non protocol-based practice was commonly used by the participants. Society of Critical Care Medicine recommended delirium assessment for all patients in the ICU through the use of a validated assessment instrument. ^4^^,^^5^ However, in our study, most doctors (76.4%) had not used any specific tool for delirium assessment and relied on signs and symptoms alone without a specific protocol. As in the study by Depetris et al., 57% of doctors did not use any specific tool for delirium assessment. ^18^ Delirium was usually assessed using either the Confusion Assessment Method for the Intensive Care Unit (CAM-ICU) or the Intensive Care Delirium Screening Checklist (ICDSC). ^7^^,^^23^ Although the usefulness of the Confusion Assessment Method for the ICU (CAM-ICU) for delirium detection has been documented in recent studies, ^5^^,^^16^ only 18.1% of our participants used CAM-ICU for delirium assessment. Nevertheless, ICDSC was used by only 1.4% of the doctors. Interestingly, in a systematic review, the CAM-ICU was the tool used for assessing delirium in 65% of delirium clinical trials, while the ICDSC was used in only 6% of the trials. ^6^ In a meta-analysis study, both the CAM-ICU and ICDSC could be used to screen for delirium and diagnose it. However, the study favored CAM-ICU as ICDSC showed lower specificity and sensitivity than CAM-ICU. ^19^ This was the case among our study participants, as only 1.4% used the ICDSC in assessing delirium, while 18.1% used the CAM-ICU tool. Comparatively, in a national multi-center study in China, 34% of doctors used delirium tools; of these, CAM-ICU was the most commonly used (83%) tool. ^22^ Nevertheless, in another study, CAM-ICU was used by 27.5% of doctors, while ICDSC was used by 5%. ^18^

Presence of delirium among ICU patients must be assessed every 8 hours or at least every 12 hours. ^7^^,^^23^ Less than half of the ICU doctors (40.3%) in our study assessed delirium every 8-12 hours, which was the practice in 60% of the clinical trials of delirium, as reported by Colantuoni et al. Furthermore, 45.8% of our doctors assessed delirium daily, which was higher than that reported (35%) by Colantuoni et al. ^6^ Only 13.9% of our doctors had no regular assessments for delirium; this was the case for 30% of physicians in a multinational study. ^18^ Among our study participants, one of the reasons for irregular delirium assessment was increased workload. This issue has been interpreted as an environmental barrier, especially among ICU staff. ^3^ This issue was addressed in a study recommended that, in such case of limited resources, delirium management strategies may be prioritized for patients with high risk of delirium. ^7^ In our study, the association between ICU doctors’ practice and their years of experience was studied. A statistically significant difference ( *p*=0.012) was found in delirium assessment. Contrary to the study by Wang et al., there was no statistically significant difference ( *p*=0.074) between experienced and less-experienced clinicians. ^22^

Delirium preventive measures are important for all ICU patients. ^7^ This was the case for most of our doctors; however, two doctors reported that they had not conducted such measures unless the patient had developed signs and symptoms of delirium.

The non-pharmacological approach to delirium management (treatment and/or prevention) is the preferred approach. ^23^ It was the first-line choice for 76.4% of the doctors in our study, which was higher than the percentage (22.5%) reported by Depetris et al. ^18^ Communication with ICU patients to prevent confusion was the most frequent nonpharmacological approach applied by ICU doctors in our study (75%). This practice involved frequent reorientation of the patients and allowing relatives to come and talk to their patients. This approach was discussed in a systematic review by Deemer et al. assessing early cognitive interventions for delirium in ICU patients. ^12^ Moreover, participation of family members in delirium prevention strategies could be complementary to the communication interventions performed by doctors and nurses. ^12^ In our study, this approach of using family member support was practiced by a single doctor. Sleep and circadian rhythm regulation among ICU patients was a targeted therapy approach for these patients ^24^; however, it was applied by only 18.1% of our doctors. Another important risk factor for delirium was the use of benzodiazepines. ^15^^,^^23^ Midazolam is the most commonly used sedative for ICU patients, prescribed as high as 72%–90.5% of sedatives. ^15^^,^^18^^,^^25^ Hence, reducing their use is considered an important non-pharmacological approach for the prevention of delirium in critically ill patients. ^23^ However, among our ICU doctors, only 6.9% adopted a reduction in benzodiazepines. Surprisingly, sedatives were used by 20% of our doctors to manage delirium and midazolam was one of the agents used by clinicians in China (31%) for treating delirium. ^22^ This issue needs to be addressed because the use of sedatives for patients with delirium worsens the case and their use should be reduced. ^16^^,^^23^

Based on a systematic review by Barbateskovic et al., evidence for the use of pharmacological interventions in the management and prevention of delirium is sparse or poor. ^26^ Nonetheless, this approach is not superior to delirium management. ^9^ The pharmacological approach for delirium treatment was used as the first-line management in 22.2% of our study participants. This was higher than reported (2.5%) in a multi-national study. ^18^ Antipsychotics were the most commonly used agents (69.4%) among our study doctors. Haloperidol was the most commonly used agent among ICU doctors (83.3%) for delirium treatment. This was consistent with a cohort study that used haloperidol alone in combination with clonidine. ^17^ Furthermore, studies have reported that haloperidol use was the highest in patients with delirium (30%, 43.3%). ^4^^,^^22^^,^^26^ The second most commonly used agent in our study was olanzapine (5.6%), similar to that reported (5.9%) by Swan et al. ^4^ In contrast, quetiapine and risperidone were used by only 1.4% of our study participants, which was much lower than the frequencies reported by Swan et al. (12.7% and 5% respectively). ^4^

Not to forget that, delirium clinically has two states; hyperactive state mostly characterized by agitation, besides the hypoactive state, ^14^ our study assessed current practice of doctors towards agitation treatment. More than half of the study participants (54.2%) prescribed antipsychotics for agitation treatment, while 33.3% prescribed sedatives. This practice of our doctors was not in line with recommended treatments, as sedatives were considered treatment agents for agitation. ^23^

Continuation of delirium medication after ICU discharge was common (50.2%), as per a previous study. ^27^ Of our doctors, 40.3% had not stopped delirium medications on discharge, which was lower than that reported above. ^27^ However, continuation of such medications beyond the hospital stay could lead to harmful and deleterious events, and medication reconciliation is crucial in such cases. ^23^

The limitations of our study were the lack of comparison of doctors practice to nurses practice. Doctors and nurses were the direct healthcare providers for critically ill patients and nurses play crucial rule in ICU practice. Involving nurses would give a complete picture about this issue. Furthermore, the data collection tool was not validated through Cronbach test of reliability. Although this study was a multi-center study, selection bias might be a risk as we focused on the Hospitals of the Military section as they were bigger and more populated hospitals.

## Conclusions

Less than half of ICU doctors assessed delirium every 8-12 hours. Non-pharmacological preventive measures were applied by the majority of participants (976.4%). Communication with patients is important for delirium prevention, as was done by most of the study participants. However, the involvement of family members in communicating with ICU patients is an important approach; yet, it is only applied by one doctor. Delirium assessment tools were used by less than 20% of the ICU doctors. Delirium may fluctuate between agitation and hypoactive states. More than half of the ICU doctors prescribed antipsychotics for the treatment of both forms. This was not the case; sedation is the preferred approach for agitated patients. Only 33.3% of the participants were prescribed sedatives to treat agitation. Medication reconciliation and contact with the next in-charge of the patients must be conducted to reduce the use of these medications after hospital discharge.

Instead of using signs and symptoms alone, ICU staff should focus on the use of delirium assessment tools. Non-pharmacological preventive measures must be implemented for all ICU patients, especially those with a high risk of developing delirium. The involvement of family members in communicating with ICU patients should be encouraged as a complementary approach to prevent delirium. A more frequent assessment of delirium is desirable among healthcare staff: 8 hours or 12 hours instead of once-daily assessment. Delirium may fluctuate between agitation and hypoactive states. Treatments for each form must be established by ICU doctors.

## Declarations

### Ethics and consent

The proposal was reviewed and approved by the Ethical Committee of Omdurman Islamic University. The ethics committee of Omdurman Islamic University reviewed and approved the proposal on 28.May.2021 after full board review (IRB name: Omdurman Islamic University Ethics Committee, Reference number: 2021/2). Approval from the Military Hospital was obtained and authorization to implement the research was granted by the administration of the ICUs. All methods were performed in accordance with the relevant guidelines and regulations of the Declarations of Helsinki ( https://www.wma.net/policies-post/wma-declaration-of-helsinki-ethical-principles-for-medical-research-involving-human-subjects/). Participants were informed about the research objectives and signed written informed consent was obtained from each participant prior to data collection. They were assured about their confidentiality through the use of an anonymous research tool and that the data collected from each of them were not to be used for any purposes other than those assigned to the research. Participants were free to accept or reject participation in this study.

## Availability of supporting data

All supporting data are available.

## Authors’ contributions

Sheema Hamid Seidna Hamid: Conceptualization, Data curation, Methodology.

Ghada Omer Hamad Abd El-Raheem: Conceptualization, Software, Formal analysis, and writing-original Draft Preparation.

Hana Eltayeb Salih Elamin: Validation, Writing-Review and Editing.

Mudawi Mohammed Ahmed Abdallah: Project adminstration, Resources.

## Data Availability

Figshare: Delirium current practice among Intensive Care Units Doctors, Khartoum- Underlying data; https://doi.org/10.6084/m9.figshare.24938505.v1. ^28^ Data are available under the terms of the Creative Commons Attribution International license (CC BY 4.0). Preprint available at: https://www.researchsquare.com/article/rs-1070778/v3.pdf DOI: https://doi.org/10.21203/rs.3.rs-1070778/v3 Methods section had provided sufficient details of the materials and methods used so that the work can be repeated by others. The section was developed based on STROBE checklist of cross-sectional studies ( https://www.equator-network.org/reporting-guidelines/strobe/).
