# Supplementary material for: Delirium management and current practice among Intensive Care Units Doctors, Khartoum
Source: F1000Res. 2025 Feb 19;13:456. Originally published 2024 May 7. [Version 4] doi: 10.12688/f1000research.142233.4 (PMC11803441; doi:10.12688/f1000research.142233.4)
Supplement: Peer review history

# Reviewer report: Reviewer response for version 4

**DOI:** 10.5256/f1000research.178238.r367580

**Published:** 2025-02-20

**Authors:** Hiroyoshi Takeuchi

**Affiliations:** 1 Keio University, Tokyo, Japan

As the authors adequately revised the manuscript according to the reviewer's comments, I have no further comments.

Is the work clearly and accurately presented and does it cite the current literature?

Yes

If applicable, is the statistical analysis and its interpretation appropriate?

Yes

Are all the source data underlying the results available to ensure full reproducibility?

Yes

Is the study design appropriate and is the work technically sound?

Yes

Are the conclusions drawn adequately supported by the results?

Yes

Are sufficient details of methods and analysis provided to allow replication by others?

Yes

Reviewer Expertise:

Schizophrenia and delirium

I confirm that I have read this submission and believe that I have an appropriate level of expertise to confirm that it is of an acceptable scientific standard.

# Reviewer report: Reviewer response for version 3

**DOI:** 10.5256/f1000research.174773.r361960

**Published:** 2025-02-06

**Authors:** Hiroyoshi Takeuchi

**Affiliations:** 1 Keio University, Tokyo, Japan

This study examines the barriers and knowledge gaps in delirium assessment and management among ICU physicians. Despite international guidelines recommending daily delirium screening, fewer than 20% of doctors utilized validated assessment tools, with their use significantly associated with clinical experience. Non-pharmacological management strategies were commonly employed, while haloperidol was the most frequently prescribed medication. Additionally, nearly half of the physicians did not discontinue delirium medications upon ICU discharge. This topic is clinically significant, the methodology is clear, and the manuscript is well-structured. I hope the following comments will help further enhance its quality:

1. Please provide the means and standard deviations for age and years of experience in Table 1.
2. Please specify what is meant by "sedatives" (e.g., benzodiazepines).
3. Please correct the spelling of “resperidone” to “risperidone.”

Is the work clearly and accurately presented and does it cite the current literature?

Yes

If applicable, is the statistical analysis and its interpretation appropriate?

Yes

Are all the source data underlying the results available to ensure full reproducibility?

Yes

Is the study design appropriate and is the work technically sound?

Yes

Are the conclusions drawn adequately supported by the results?

Yes

Are sufficient details of methods and analysis provided to allow replication by others?

Yes

Reviewer Expertise:

Schizophrenia and delirium

I confirm that I have read this submission and believe that I have an appropriate level of expertise to confirm that it is of an acceptable scientific standard, however I have significant reservations, as outlined above.

# Response

**Published:** 2025-02-12

**Authors:** Ghada Abd El-Raheem

**Affiliations:** Imperial Hospital, Soba University Hospital, Military Hospital, Sudan

Dear Prof. Hiroyoshi Takeuchi,

 Thank you for devoting your time for reviewing our paper.

 All your amendments were fulfilled as follows:

 1. Age and years of experience were expressed as mean and SD.

 2. Specified sedatives as benzodiazepines.

 3. Corrected the spelling of Risperidone in the text and figure.

 Thank you again

 Best regards, 

 Corresponding Author

# Reviewer report: Reviewer response for version 3

**DOI:** 10.5256/f1000research.174773.r354246

**Published:** 2025-01-24

**Authors:** Helmut Frohnhofen

**Affiliations:** 1 University Hospital Düsseldorf, Düsseldorf, Germany

Thank you for inviting me to review the paper: Delirium management and current practice among Intensive Care Units Doctors, Khartoum.

 The study is a hospital based cross sectional study. The investigators selected randomly 72 ICU physicians.

 The results show that most physicians are younger (<30 years of age). Validated tools to detect delirium are rarely applied. Treatment for delirium is often conducted by haloperiodol and this drug is continued at discharge from the ICU. 

 The study is well designed and conducted. Analysis is adequately done.

 The conclusion is convincing and recommend to use validated tool for detection of delirium in ICU patients more often.

 My decision: accept as it is.

Is the work clearly and accurately presented and does it cite the current literature?

Yes

If applicable, is the statistical analysis and its interpretation appropriate?

Yes

Are all the source data underlying the results available to ensure full reproducibility?

Yes

Is the study design appropriate and is the work technically sound?

Yes

Are the conclusions drawn adequately supported by the results?

No

Are sufficient details of methods and analysis provided to allow replication by others?

Yes

Reviewer Expertise:

Internal medicine, intensive care

I confirm that I have read this submission and believe that I have an appropriate level of expertise to confirm that it is of an acceptable scientific standard.

# Reviewer report: Reviewer response for version 2

**DOI:** 10.5256/f1000research.172723.r336635

**Published:** 2024-11-12

**Authors:** Richard E Kennedy

**Affiliations:** 1 University of Alabama at Birmingham, Birmingham, USA

This is a revision of a manuscript examining the barriers and gaps in knowledge about treatment of delirium among ICU physicians in Sudan.  The authors have generally responded well to the previous concerns,  providing additional details on the setting and questionnaires used in the study; the justification for examining this topic specifically in Sudan; and the potential limitations.

 The remaining concern I would have is that Reference 18, which is cited in the text as an international guideline, is for patients with burns rather than patients in the ICU, and it is not clear how well these recommendations would apply to the ICU population.  The authors state in their response that this has been corrected but it is still a reference to burn patients; it would seem a guideline for the ICU should be cited instead.

Is the work clearly and accurately presented and does it cite the current literature?

Partly

If applicable, is the statistical analysis and its interpretation appropriate?

Partly

Are all the source data underlying the results available to ensure full reproducibility?

Yes

Is the study design appropriate and is the work technically sound?

Partly

Are the conclusions drawn adequately supported by the results?

Partly

Are sufficient details of methods and analysis provided to allow replication by others?

No

Reviewer Expertise:

Delirium phenomenology, diagnosis, and clinical trials

I confirm that I have read this submission and believe that I have an appropriate level of expertise to confirm that it is of an acceptable scientific standard, however I have significant reservations, as outlined above.

# Response

**Published:** 2024-11-12

**Authors:** Ghada Abd El-Raheem

**Affiliations:** Imperial Hospital, Soba University Hospital, Military Hospital, Sudan

Dear reviewer, 

 Thank you for your valuable time and comments. We revised the references and corrected the reference 18. Thank you again.

# Reviewer report: Reviewer response for version 1

**DOI:** 10.5256/f1000research.155756.r295363

**Published:** 2024-07-29

**Authors:** Richard E Kennedy

**Affiliations:** 1 University of Alabama at Birmingham, Birmingham, USA

This manuscript reports on the barriers and gaps in knowledge about delirium among a sample of ICU physicians in Sudan.  A random selection of providers were queried regarding their knowledge and practice in management of delirium, which were summarized and related to provider characteristics. The authors found that formal assessment of delirium was uncommon.  Nonpharmacological management was commonly used, but pharmacological management was also frequent.

 The topic is potentially an important one, as difficulties recognizing delirium are common across a number of settings, and are associated with a number of adverse patient and healthcare system outcomes. However, the methods are often not specified in sufficient detail, and the contribution of this particular study to the existing literature is not well delineated.

 The background is generally well done, but a bit disorganized in a few spots.  In particular, the statement "Furthermore, delirium is associated with long-term cognitive impairments." seems out of placer from the rest of the material in the paragraph, and would be better placed with other material about adverse consequences.  Similarly, the statement "From another perspective, patients with delirium are more likely to bear increased healthcare costs than those without delirium." could be combined with material about adverse consequences rather than a separate paragraph.  Reference 18 is for burn patients and it is not clear that these findings are applicable to the ICU setting.  And noting that the mixed form of delirium is actually the most common would be helpful.

 Most importantly, the background does not clearly lay out the case for the current study.  There have been multiple publications in the literature noting that delirium is often missed by clinicians, that assessment is often not performed, and many clinicians lack sufficient understanding of delirium.  Given these previous studies, why is this study needed?  Are there particular aspects of practice in Sudan that are different from previous studies?  If so, this should be more clearly delineated to show the importance of this investigation.

 Several aspects of the methods are not sufficiently described.  The setting notes several different types of ICUs being studied but these are not delineated.  The stratified random sampling needs to be described more fully.  The questionnaire used for assessing knowledge and practice is not well described - was this a standard questionnaire (in which case it should be referenced)?  Or a questionnaire developed by the investigators (in which case the questions should be provided in detail, along with the validation of the questionnaire for adequately assessing the outcomes of interest)? Why were the predictor variables selected, and are these 4 sufficient for describing the associations in the sample?  Is the chosen sample size formula appropriate for a series of chi-square tests?  

 The results are generally straightforward.  However, in the description of the sample, did all of the randomized participants complete the questionnaire?  Or were there refusals?  A description of the flow of participants (or a flow diagram, if it is complicated) would be helpful. In addition, the sample was overwhelmingly young, inexperienced, and female.  Is this typical of ICU doctors in Sudan?  Or were there biases in the sample?

 When an ICU doctor determined delirium based on "signs and symptoms", does this mean that DSM-5 (or a comparable system) was used?  Or does this mean an informal assessment with no specific criteria?

 Similarly, what does "communication with the patient to prevent confusion" mean?  Does this mean established protocols such as frequent reorientation, or is this an informal communication without a specific protocol?  Such information is helpful in being able to understand and replicate the study findings.

 It would be helpful to present odds ratios from the chi-square tests to indicate the direction of the associations, rather than just p values.

 The discussion would benefit from more depth.  There is a rather extensive literature on the recognition of delirium by physicians, the barriers to recognition, and attitudes towards management.  However, the discussion only references a few studies (often a comparison to just 1 other study) rather than providing an overview of previous findings.  Furthermore, the authors often only compare their results to a previous study, but do not place this in context - what does it mean if their results are similar or different from previous studies?  Does this have implications for the recognition and management of delirium generally, or does this show specific aspects of management of delirium in Sudanese hospitals?

 Under limitations, the authors should not just note the limitations, but also how these impact the study - do these affect the reliability or generalizability of the findings?  Also under limitations, is the military hospital setting a limitation?  Are the patient characteristics for military hospitals significantly different from the patient characteristics in non-military hospitals in Sudan, the way that there are differences in other countries?

 Overall, I think that this manuscript has the potential to make a contribution to the literature, particularly since delirium and other cognitive disorders are not well studied in settings outside of a few developed countries.  However, more details are needed in several places to fully understand the study, be able to replicate their processes, and place the findings in context.

Is the work clearly and accurately presented and does it cite the current literature?

Partly

If applicable, is the statistical analysis and its interpretation appropriate?

Partly

Are all the source data underlying the results available to ensure full reproducibility?

Yes

Is the study design appropriate and is the work technically sound?

Partly

Are the conclusions drawn adequately supported by the results?

Partly

Are sufficient details of methods and analysis provided to allow replication by others?

No

Reviewer Expertise:

Delirium phenomenology, diagnosis, and clinical trials

I confirm that I have read this submission and believe that I have an appropriate level of expertise to state that I do not consider it to be of an acceptable scientific standard, for reasons outlined above.

# Response

**Published:** 2024-09-29

**Authors:** Ghada Abd El-Raheem

**Affiliations:** Imperial Hospital, Soba University Hospital, Military Hospital, Sudan

Dear reviewer Richard Kennedy,

 Thank you for your valuable time and comments. We revised all your comments and fulfilled them.

 The introduction was corrected and rephrased and ref. describing burn patients is changed. As well as, the aim was well described.

 The methods section is detailed to be more reproducible. the details of the sampling process and the questionnaire are all detailed and provided reference for the questions asked. Also differences in the study population between military and civilian hospitals was detailed. Types of patients in military hospitals were described.

 Risk estimate analysis can not be computed, they are only computed for 2x2 tables without empty cells.

 Discussion was rewritten to be more in depth and showing all the aspects of delirium management among Sudanese doctors.

 Limitations were adjusted to detail the impact of limitations on the reliability and generalizability.
